# Poly[[dodeca­aqua­(μ_4_-benzene-1,4-dicarboxyl­ato)(μ_2_-4,4′-bipyridine-κ^2^ *N*:*N*′)dicerium(III)] bis­(benzene-1,4-dicarboxyl­ate)]

**DOI:** 10.1107/S1600536812016388

**Published:** 2012-04-21

**Authors:** Hitoshi Kumagai, Yoshiyuki Sakamoto, Satoshi Kawata, Shinji Inagaki

**Affiliations:** a Toyota Central R and D Labs. Inc., Nagakute 41-1, Aichi, Japan; b Department of Chemistry, Fukuoka University, Fukuoka 814-0180, Japan

## Abstract

The asymmetric unit of the title compound, {[Ce_2_(C_8_H_4_O_4_)(C_10_H_8_N_2_)(H_2_O)_12_](C_8_H_4_O_4_)_2_}_*n*_, consists of half a Ce^III^ cation, a quarter of a coordinated benzene-1,4-dicarboxyl­ate (bdc^2−^) dianion, a quarter of a 4,4′-bipyridine (bpy) mol­ecule, three water mol­ecules and a half of an uncoordinated benzene-1,4-dicarboxyl­ate dianion. The Ce^III^ ion is located on a twofold rotation axis and exhibits a distorted trigonal prism square-face tricapped coordination geometry. The coordinated and uncoordinated bdc^2−^ ions and the bpy mol­ecule lie about special positions of site symmetries 2/*m*, *m* and 2/*m*, respectively. The Ce^III^ ions are bridged by the bdc^2−^ and bpy ligands, giving a sheet structure parallel to the *ac* plane. The uncoordinated bdc^2−^ dianion exists between the sheets and links the sheets by inter­molecular O—H⋯O hydrogen bonds between the uncoordinated bdc^2−^ and coordinated water mol­ecules. A π–π stacking inter­action between the uncoordinated bdc^2−^ dianion and the bpy ligand [centroid–centroid distance = 3.750 (4) Å] is also observed.

## Related literature

For coordination polymers, see: Cheetham *et al.* (1999 ▶); Furukawa *et al.* (2010 ▶). For related host–guest systems, see: Kawata & Kitagawa (2002 ▶).
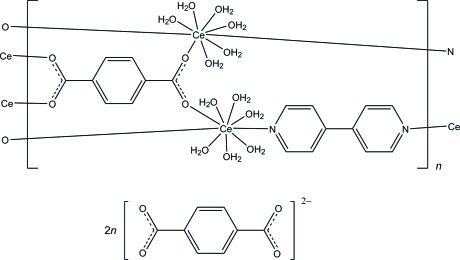


## Experimental

### 

#### Crystal data

[Ce_2_(C_8_H_4_O_4_)(C_10_H_8_N_2_)(H_2_O)_12_](C_8_H_4_O_4_)_2_
*M* *_r_* = 572.48
Orthorhombic, 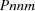

*a* = 6.112 (4) Å
*b* = 14.278 (8) Å
*c* = 22.395 (12) Å
*V* = 1954.3 (18) Å^3^
*Z* = 4
Mo *K*α radiation
μ = 2.40 mm^−1^
*T* = 293 K
0.60 × 0.20 × 0.10 mm

#### Data collection

Rigaku Mercury70 diffractometer
Absorption correction: multi-scan (*REQAB*; Rigaku, 1998 ▶) *T*_min_ = 0.511, *T*_max_ = 0.787
14945 measured reflections
2246 independent reflections
2163 reflections with *F* ^2^ > 2σ(*F* ^2^)
*R*_int_ = 0.029

#### Refinement

*R*[*F* ^2^ > 2σ(*F* ^2^)] = 0.034
*wR*(*F* ^2^) = 0.068
*S* = 1.39
2246 reflections
167 parameters
6 restraints
H atoms treated by a mixture of independent and constrained refinement
Δρ_max_ = 0.49 e Å^−3^
Δρ_min_ = −0.42 e Å^−3^

### 

Data collection: *CrystalClear* (Rigaku/MSC, 2005 ▶); cell refinement: *CrystalClear*; data reduction: *CrystalClear*; program(s) used to solve structure: *SIR2008* (Burla *et al.*, 2007 ▶); program(s) used to refine structure: *SHELXL97* (Sheldrick, 2008 ▶); molecular graphics: *CrystalStructure* (Rigaku, 2010 ▶); software used to prepare material for publication: *CrystalStructure*.

## Supplementary Material

Crystal structure: contains datablock(s) global, I. DOI: 10.1107/S1600536812016388/is5110sup1.cif

Structure factors: contains datablock(s) I. DOI: 10.1107/S1600536812016388/is5110Isup3.hkl

Supplementary material file. DOI: 10.1107/S1600536812016388/is5110Isup4.cdx

Additional supplementary materials:  crystallographic information; 3D view; checkCIF report

## Figures and Tables

**Table 1:** Selected bond lengths (Å)

Ce1—O1	2.479 (3)
Ce1—O1^i^	2.479 (3)
Ce1—O2	2.530 (3)
Ce1—O2^i^	2.530 (3)
Ce1—O3	2.551 (3)
Ce1—O3^i^	2.551 (3)
Ce1—O4	2.533 (3)
Ce1—O4^i^	2.533 (3)
Ce1—N1	2.873 (5)

Symmetry code: (i) 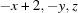.

**Table 2:** Hydrogen-bond geometry (Å, °)

*D*—H⋯*A*	*D*—H	H⋯*A*	*D*⋯*A*	*D*—H⋯*A*
O2—H2⋯O5	0.84 (4)	1.93 (4)	2.754 (5)	167 (5)
O2—H3⋯O6^ii^	0.84 (3)	1.90 (4)	2.725 (5)	166 (4)
O3—H4⋯O5	0.83 (5)	2.02 (5)	2.828 (4)	164 (6)
O3—H5⋯O6^iii^	0.84 (5)	1.81 (5)	2.650 (4)	175 (6)
O4—H7⋯O5^iv^	0.84 (4)	1.91 (4)	2.749 (5)	174 (6)

Symmetry codes: (ii) 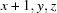; (iii) 
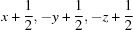
; (iv) 
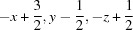
.

**Fig. 1.**
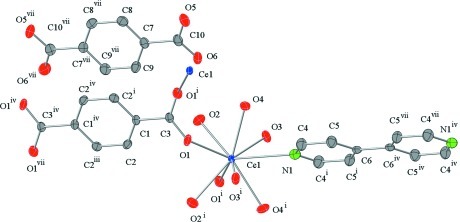
View of the title compound with atomic numbering scheme. Hydrogen atoms have been omitted for clarity. [Symmetry codes: (i) -x + 2, -y, z; (iii) x, y, -z + 1; (iv) -x + 2, -y, -z; (vii) x, y, -z.]

**Fig. 2.**
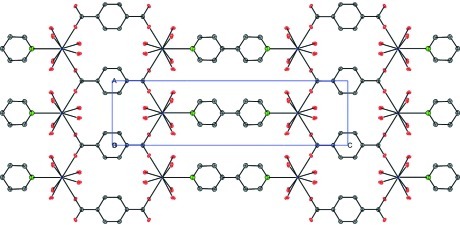
Packing diagram of the title compound, showing a sheet structure. Hydrogen atoms have been omitted for clarity.

**Fig. 3.**
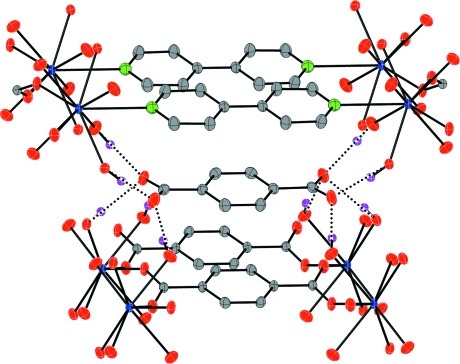
Hydrogen bonding interactions for the title compound. Hydrogen atoms and hydrogen bonding interactions are shown as purple color and dashed line, respectively.

## supplementary crystallographic information

### Comment

The design of coordination polymers (CPs), also known as metal-organic frameworks (MOFs), have received considerable attention in recent years due to potential applications for sorption, catalysis, optical, magnetic materials and host-guest interactions (Cheetham *et al.*, 1999; Furukawa *et al.*, 2010; Kawata & Kitagawa, 2002). Here we report synthesis and single-crystal structure of the title compound.

The coordination polymer of the title compound, {[Ce_2_(C_8_H_4_O_4_)(C_10_H_10_N_2_)(H_2_O)_12_](C_8_H_4_O_4_)_2_}*_n_*, consists of Ce^III^ ions, bdc^2-^ dianions and bpy as bridging ligands, and water molecules. In the crystal, two types of bdc^2-^ dianions are found. One bdc^2-^ dianion coordinates to Ce^III^ ions and acts as a bridging ligand to form a two-dimensional network. The other is an uncoordinated bdc^2-^ dianion. Uncoordinated bdc^2-^ dianions are stabilized by intermolecular hydrogen bonds between the uncoordinated bdc^2-^ and coordinated water molecules and π–π stacking interactions between uncoordinated bdc^2-^ dianions and bridging ligands to give a three-dimensional network structure.

### Experimental

An aqueous solution (5 ml) of cerium nitrate hexahydrate (0.81 g) was transferred to a glass tube, then an ethanol-water mixture (5 ml) of tetrabromoerephthalic acid (0.2 g), NaOH (0.08 g) and 4,4'-bpy (0.19 g) was poured into the glass tube without mixing the two solutions. Colorless crystals began to form at ambient temperature in 1 month. One of these crystals was used for X-ray crystallography.

### Refinement

H atoms bonded to C atoms were introduced at the positions calculated theoretically (C—H = 0.93 Å) and treated as riding, with *U*_iso_(H) = 1.2*U*_eq_(C). H atoms of water molecules were located in a difference Fourier map and were refined isotropically with distance restraints of O—H = 0.84 (2) Å.

### Crystal data

**Table:**

[Ce_2_(C_8_H_4_O_4_)(C_10_H_8_N_2_)(H_2_O)_12_](C_8_H_4_O_4_)_2_	*F*(000) = 1140.00
*M**_r_* = 572.48	*D*_x_ = 1.946 Mg m^−^^3^
Orthorhombic, *P**n**n**m*	Mo *K*α radiation, λ = 0.71070 Å
Hall symbol: -P 2 2n	Cell parameters from 3992 reflections
*a* = 6.112 (4) Å	θ = 3.1–27.5°
*b* = 14.278 (8) Å	µ = 2.40 mm^−^^1^
*c* = 22.395 (12) Å	*T* = 293 K
*V* = 1954.3 (18) Å^3^	Platelet, colorless
*Z* = 4	0.60 × 0.20 × 0.10 mm

### Data collection

**Table:**

Rigaku Mercury70 diffractometer	2163 reflections with *F*^2^ > 2σ(*F*^2^)
Detector resolution: 7.314 pixels mm^-1^	*R*_int_ = 0.029
ω scans	θ_max_ = 27.5°
Absorption correction: multi-scan (*REQAB*; Rigaku, 1998)	*h* = −7→7
*T*_min_ = 0.511, *T*_max_ = 0.787	*k* = −18→18
14945 measured reflections	*l* = −29→29
2246 independent reflections	

### Refinement

**Table:**

Refinement on *F*^2^	Secondary atom site location: difference Fourier map
*R*[*F*^2^ > 2σ(*F*^2^)] = 0.034	Hydrogen site location: inferred from neighbouring sites
*wR*(*F*^2^) = 0.068	H atoms treated by a mixture of independent and constrained refinement
*S* = 1.39	*w* = 1/[σ^2^(*F*_o_^2^) + (0.*P*)^2^ + 7.6254*P*] where *P* = (*F*_o_^2^ + 2*F*_c_^2^)/3
2246 reflections	(Δ/σ)_max_ < 0.001
167 parameters	Δρ_max_ = 0.49 e Å^−^^3^
6 restraints	Δρ_min_ = −0.42 e Å^−^^3^
Primary atom site location: structure-invariant direct methods	

### Special details

**Table:**

Geometry. ENTER SPECIAL DETAILS OF THE MOLECULAR GEOMETRY
Refinement. Refinement was performed using all reflections. The weighted *R*-factor (*wR*) and goodness of fit (*S*) are based on *F*^2^. *R*-factor (σt) are based on *F*. The threshold expression of *F*^2^ > 2.0 σ(*F*^2^) is used only for calculating *R*-factor (σt).

### Fractional atomic coordinates and isotropic or equivalent isotropic displacement parameters (Å^2^)

**Table:**

	*x*	*y*	*z*	*U*_iso_*/*U*_eq_	
Ce1	1.0000	0.0000	0.286968 (11)	0.01450 (9)	
O1	0.6621 (5)	0.03486 (19)	0.34397 (11)	0.0229 (6)	
O2	1.0784 (5)	0.1316 (2)	0.35957 (13)	0.0257 (6)	
O3	0.8063 (5)	0.14579 (19)	0.24774 (12)	0.0234 (6)	
O4	0.6817 (5)	−0.0885 (2)	0.24157 (13)	0.0259 (6)	
O5	0.7801 (5)	0.2740 (2)	0.34373 (11)	0.0276 (7)	
O6	0.4224 (6)	0.2508 (3)	0.34529 (12)	0.0331 (8)	
N1	1.0000	0.0000	0.15870 (19)	0.0230 (10)	
C1	0.5000	0.0000	0.4378 (2)	0.0152 (9)	
C2	0.6884 (6)	0.0245 (3)	0.46904 (14)	0.0171 (8)	
C3	0.5000	0.0000	0.37044 (19)	0.0152 (9)	
C4	0.8241 (7)	0.0259 (3)	0.12810 (16)	0.0252 (9)	
C5	0.8174 (7)	0.0271 (3)	0.06620 (16)	0.0244 (9)	
C6	1.0000	0.0000	0.0334 (2)	0.0187 (10)	
C7	0.6065 (7)	0.2588 (3)	0.43811 (15)	0.0182 (7)	
C8	0.7943 (7)	0.2825 (3)	0.46897 (16)	0.0209 (8)	
C9	0.4180 (7)	0.2347 (3)	0.46905 (15)	0.0218 (8)	
C10	0.6024 (7)	0.2610 (3)	0.37083 (16)	0.0214 (8)	
H1	0.8145	0.0410	0.4483	0.0206*	
H2	0.983 (7)	0.174 (3)	0.361 (3)	0.041 (15)*	
H3	1.197 (5)	0.160 (3)	0.353 (2)	0.030 (14)*	
H4	0.778 (11)	0.188 (4)	0.272 (2)	0.07 (2)*	
H5	0.848 (10)	0.176 (4)	0.2178 (18)	0.062 (19)*	
H6	0.555 (5)	−0.094 (5)	0.255 (3)	0.059 (19)*	
H7	0.684 (10)	−0.130 (3)	0.2149 (18)	0.053 (17)*	
H8	0.6998	0.0439	0.1491	0.0303*	
H9	0.6908	0.0461	0.0466	0.0293*	
H10	0.9207	0.2984	0.4482	0.0251*	
H11	0.2917	0.2186	0.4483	0.0262*	

### Atomic displacement parameters (Å^2^)

**Table:**

	*U*^11^	*U*^22^	*U*^33^	*U*^12^	*U*^13^	*U*^23^
Ce1	0.01572 (15)	0.01786 (15)	0.00993 (13)	−0.00118 (11)	0.0000	0.0000
O1	0.0214 (14)	0.0323 (15)	0.0151 (12)	−0.0021 (12)	0.0063 (11)	−0.0001 (11)
O2	0.0233 (15)	0.0241 (15)	0.0296 (15)	0.0009 (13)	−0.0026 (13)	−0.0080 (12)
O3	0.0324 (17)	0.0219 (14)	0.0160 (13)	0.0043 (13)	0.0037 (12)	0.0031 (11)
O4	0.0189 (15)	0.0319 (16)	0.0269 (15)	−0.0084 (13)	0.0037 (12)	−0.0094 (12)
O5	0.0305 (17)	0.0330 (16)	0.0191 (13)	0.0052 (14)	0.0063 (12)	0.0056 (11)
O6	0.0366 (18)	0.0413 (18)	0.0215 (14)	−0.0084 (15)	−0.0094 (13)	0.0093 (13)
N1	0.024 (3)	0.022 (3)	0.023 (2)	−0.004 (3)	0.0000	0.0000
C1	0.017 (3)	0.017 (3)	0.011 (2)	0.001 (3)	0.0000	0.0000
C2	0.0144 (18)	0.0205 (19)	0.0165 (17)	−0.0018 (14)	0.0031 (14)	0.0010 (13)
C3	0.020 (3)	0.014 (3)	0.012 (2)	0.002 (3)	0.0000	0.0000
C4	0.023 (2)	0.033 (3)	0.0206 (18)	0.0004 (17)	0.0021 (15)	−0.0021 (15)
C5	0.023 (2)	0.031 (3)	0.0193 (18)	0.0004 (17)	−0.0021 (15)	−0.0005 (15)
C6	0.025 (3)	0.015 (3)	0.016 (3)	−0.009 (3)	0.0000	0.0000
C7	0.0205 (19)	0.0173 (18)	0.0167 (16)	0.0034 (15)	0.0002 (14)	0.0015 (13)
C8	0.017 (2)	0.0222 (19)	0.0233 (19)	−0.0012 (16)	0.0033 (15)	0.0021 (15)
C9	0.0181 (19)	0.027 (2)	0.0199 (18)	0.0009 (16)	−0.0045 (15)	0.0000 (15)
C10	0.029 (2)	0.0168 (18)	0.0184 (17)	0.0037 (16)	−0.0012 (15)	0.0026 (14)

### Geometric parameters (Å, º)

**Table:**

Ce1—O1	2.479 (3)	C5—C6	1.391 (5)
Ce1—O1^i^	2.479 (3)	C6—C6^iv^	1.496 (7)
Ce1—O2	2.530 (3)	C7—C8	1.382 (6)
Ce1—O2^i^	2.530 (3)	C7—C9	1.388 (6)
Ce1—O3	2.551 (3)	C7—C10	1.507 (5)
Ce1—O3^i^	2.551 (3)	C8—C8^iii^	1.390 (6)
Ce1—O4	2.533 (3)	C9—C9^iii^	1.386 (5)
Ce1—O4^i^	2.533 (3)	O2—H2	0.84 (4)
Ce1—N1	2.873 (5)	O2—H3	0.84 (4)
O1—C3	1.257 (4)	O3—H4	0.83 (5)
O5—C10	1.258 (5)	O3—H5	0.84 (5)
O6—C10	1.248 (5)	O4—H6	0.84 (4)
N1—C4	1.327 (5)	O4—H7	0.84 (4)
N1—C4^i^	1.327 (5)	C2—H1	0.930
C1—C2	1.392 (5)	C4—H8	0.930
C1—C2^ii^	1.392 (5)	C5—H9	0.930
C1—C3	1.509 (7)	C8—H10	0.930
C2—C2^iii^	1.387 (5)	C9—H11	0.930
C4—C5	1.387 (6)		
			
C5···C7^v^	3.532 (6)	C6···C8^vi^	3.589 (4)
C5···C9^v^	3.544 (6)	C7···C2	3.453 (6)
C6···C7^v^	3.562 (4)	C7···C5^vii^	3.532 (6)
C6···C7^vi^	3.562 (4)	C7···C6^vii^	3.562 (4)
C6···C8^v^	3.589 (4)	C8···C6^vii^	3.589 (4)
			
O1—Ce1—O1^i^	118.00 (9)	C2—C1—C2^ii^	119.7 (4)
O1—Ce1—O2	71.20 (10)	C2—C1—C3	120.2 (2)
O1—Ce1—O2^i^	70.15 (10)	C2^ii^—C1—C3	120.2 (2)
O1—Ce1—O3	68.08 (10)	C1—C2—C2^iii^	120.2 (4)
O1—Ce1—O3^i^	136.72 (9)	O1—C3—O1^ii^	123.7 (4)
O1—Ce1—O4	70.53 (10)	O1—C3—C1	118.1 (2)
O1—Ce1—O4^i^	138.31 (10)	O1^ii^—C3—C1	118.1 (2)
O1—Ce1—N1	121.00 (7)	N1—C4—C5	122.9 (4)
O1^i^—Ce1—O2	70.15 (10)	C4—C5—C6	120.1 (4)
O1^i^—Ce1—O2^i^	71.20 (10)	C5—C6—C5^i^	116.2 (4)
O1^i^—Ce1—O3	136.72 (9)	C5—C6—C6^iv^	121.9 (3)
O1^i^—Ce1—O3^i^	68.08 (10)	C5^i^—C6—C6^iv^	121.9 (3)
O1^i^—Ce1—O4	138.31 (10)	C8—C7—C9	120.0 (4)
O1^i^—Ce1—O4^i^	70.53 (10)	C8—C7—C10	120.6 (4)
O1^i^—Ce1—N1	121.00 (7)	C9—C7—C10	119.4 (4)
O2—Ce1—O2^i^	100.03 (11)	C7—C8—C8^iii^	120.0 (4)
O2—Ce1—O3	72.74 (10)	C7—C9—C9^iii^	120.0 (4)
O2—Ce1—O3^i^	137.67 (10)	O5—C10—O6	123.9 (4)
O2—Ce1—O4	140.68 (10)	O5—C10—C7	118.1 (4)
O2—Ce1—O4^i^	75.06 (10)	O6—C10—C7	118.1 (4)
O2—Ce1—N1	129.99 (7)	Ce1—O2—H2	115 (3)
O2^i^—Ce1—O3	137.67 (10)	Ce1—O2—H3	114 (3)
O2^i^—Ce1—O3^i^	72.74 (10)	H2—O2—H3	104 (4)
O2^i^—Ce1—O4	75.06 (10)	Ce1—O3—H4	117 (4)
O2^i^—Ce1—O4^i^	140.68 (10)	Ce1—O3—H5	124 (4)
O2^i^—Ce1—N1	129.99 (7)	H4—O3—H5	102 (5)
O3—Ce1—O3^i^	139.71 (9)	Ce1—O4—H6	128 (4)
O3—Ce1—O4	84.96 (10)	Ce1—O4—H7	128 (4)
O3—Ce1—O4^i^	79.12 (10)	H6—O4—H7	102 (6)
O3—Ce1—N1	69.85 (7)	C1—C2—H1	119.897
O3^i^—Ce1—O4	79.12 (10)	C2^iii^—C2—H1	119.930
O3^i^—Ce1—O4^i^	84.96 (10)	N1—C4—H8	118.556
O3^i^—Ce1—N1	69.85 (7)	C5—C4—H8	118.540
O4—Ce1—O4^i^	132.66 (10)	C4—C5—H9	119.957
O4—Ce1—N1	66.33 (7)	C6—C5—H9	119.991
O4^i^—Ce1—N1	66.33 (7)	C7—C8—H10	119.995
Ce1—O1—C3	145.08 (19)	C8^iii^—C8—H10	119.992
Ce1—N1—C4	121.1 (3)	C7—C9—H11	120.023
Ce1—N1—C4^i^	121.1 (3)	C9^iii^—C9—H11	120.026
C4—N1—C4^i^	117.8 (4)		
			
O1—Ce1—O1^i^—C3^viii^	−84.2 (3)	O4^i^—Ce1—N1—C4^i^	51.94 (8)
O1^i^—Ce1—O1—C3	−84.2 (3)	Ce1—O1—C3—O1^ii^	−75.3 (4)
O2—Ce1—O1—C3	−138.3 (3)	Ce1—O1—C3—C1	104.7 (4)
O2^i^—Ce1—O1—C3	−29.7 (3)	Ce1—N1—C4—C5	179.8 (2)
O3—Ce1—O1—C3	143.3 (4)	Ce1—N1—C4^i^—C5^i^	179.8 (2)
O3^i^—Ce1—O1—C3	2.8 (4)	C4—N1—C4^i^—C5^i^	−0.2 (5)
O4—Ce1—O1—C3	50.9 (3)	C4^i^—N1—C4—C5	−0.2 (5)
O4^i^—Ce1—O1—C3	−175.9 (3)	C2—C1—C2^ii^—C2^ix^	0.0 (4)
O1—Ce1—N1—C4	5.40 (8)	C2^ii^—C1—C2—C2^iii^	0.0 (4)
O1—Ce1—N1—C4^i^	−174.60 (8)	C2—C1—C3—O1	−9.77 (16)
N1—Ce1—O1—C3	95.8 (3)	C2—C1—C3—O1^ii^	170.23 (16)
O2—Ce1—O1^i^—C3^viii^	−29.7 (3)	C2^ii^—C1—C3—O1	170.23 (16)
O2^i^—Ce1—O1^i^—C3^viii^	−138.3 (3)	C2^ii^—C1—C3—O1^ii^	−9.77 (16)
O3—Ce1—O1^i^—C3^viii^	2.8 (4)	C1—C2—C2^iii^—C1^ix^	0.0 (5)
O3^i^—Ce1—O1^i^—C3^viii^	143.3 (4)	N1—C4—C5—C6	0.4 (6)
O4—Ce1—O1^i^—C3^viii^	−175.9 (3)	C4—C5—C6—C5^i^	−0.2 (4)
O4^i^—Ce1—O1^i^—C3^viii^	50.9 (3)	C4—C5—C6—C6^iv^	179.8 (3)
O1^i^—Ce1—N1—C4	−174.60 (8)	C5—C6—C6^iv^—C5^iv^	180.00 (19)
O1^i^—Ce1—N1—C4^i^	5.40 (8)	C5—C6—C6^iv^—C5^x^	0.00 (19)
N1—Ce1—O1^i^—C3^viii^	95.8 (3)	C5^i^—C6—C6^iv^—C5^iv^	0.00 (19)
O2—Ce1—N1—C4	−85.35 (10)	C8—C7—C9—C9^iii^	0.2 (6)
O2—Ce1—N1—C4^i^	94.65 (10)	C9—C7—C8—C8^iii^	−0.2 (6)
O2^i^—Ce1—N1—C4	94.65 (10)	C8—C7—C10—O5	8.2 (6)
O2^i^—Ce1—N1—C4^i^	−85.35 (10)	C8—C7—C10—O6	−170.9 (4)
O3—Ce1—N1—C4	−41.42 (8)	C10—C7—C8—C8^iii^	178.4 (3)
O3—Ce1—N1—C4^i^	138.58 (8)	C9—C7—C10—O5	−173.2 (4)
O3^i^—Ce1—N1—C4	138.58 (8)	C9—C7—C10—O6	7.6 (5)
O3^i^—Ce1—N1—C4^i^	−41.42 (8)	C10—C7—C9—C9^iii^	−178.4 (3)
O4—Ce1—N1—C4	51.94 (8)	C7—C8—C8^iii^—C7^iii^	0.0 (6)
O4—Ce1—N1—C4^i^	−128.06 (8)	C7—C9—C9^iii^—C7^iii^	0.0 (6)
O4^i^—Ce1—N1—C4	−128.06 (8)		

Symmetry codes: (i) −*x*+2, −*y*, *z*; (ii) −*x*+1, −*y*, *z*; (iii) *x*, *y*, −*z*+1; (iv) −*x*+2, −*y*, −*z*; (v) *x*+1/2, −*y*+1/2, −*z*+1/2; (vi) −*x*+3/2, *y*−1/2, −*z*+1/2; (vii) *x*−1/2, −*y*+1/2, −*z*+1/2; (viii) *x*+1, *y*, *z*; (ix) −*x*+1, −*y*, −*z*+1; (x) *x*, *y*, −*z*.

### Hydrogen-bond geometry (Å, º)

**Table:**

*D*—H···*A*	*D*—H	H···*A*	*D*···*A*	*D*—H···*A*
O2—H2···O5	0.84 (4)	1.93 (4)	2.754 (5)	167 (5)
O2—H3···O6^viii^	0.84 (3)	1.90 (4)	2.725 (5)	166 (4)
O3—H4···O5	0.83 (5)	2.02 (5)	2.828 (4)	164 (6)
O3—H5···O6^v^	0.84 (5)	1.81 (5)	2.650 (4)	175 (6)
O4—H7···O5^vi^	0.84 (4)	1.91 (4)	2.749 (5)	174 (6)

Symmetry codes: (v) *x*+1/2, −*y*+1/2, −*z*+1/2; (vi) −*x*+3/2, *y*−1/2, −*z*+1/2; (viii) *x*+1, *y*, *z*.
